# Retraction Note: circRNA hsa_circRNA_0000069 promotes the proliferation, migration and invasion of cervical cancer through miR-873-5p/TUSC3 axis

**DOI:** 10.1186/s12935-023-02964-0

**Published:** 2023-06-13

**Authors:** Shuaisai Zhang, Zhengli Chen, Jinxue Sun, Na An, Qinghua Xi

**Affiliations:** 1 Department of Gynecology, Affiliated Maternity and Child Health Care Hospital of Nantong University, Nantong, Jiangsu 226018 China; 2 The laboratory department of The First Hospital of Hebei Medical University, No. 89 Donggang Road, Shijiazhuang, Hebei 050030 China; 3 Clinical Laboratory Shandong Provincial Third Hospital, Jinan, Shandong 250031 China; 4 Department of Gynecology, Shengli Oilfield Central Hospital, No. 31, Jinan Road, Dongying, Shandong 257034 China; 5 Department of Gynecology, Affiliated Hospital of Nantong University, Nantong, Jiangsu 226001 China

Cancer Cell International (2020) 20:287

10.1186/s12935-020-01387-5

The Editors-in-Chief have retracted this article. Concerns were raised regarding Fig. 2d. This figure appears to overlap with Fig. 4f in an article by different authors that was simultaneously under consideration with another journal [1]. The Editors-in-Chief therefore no longer have confidence in the results and conclusions of this article.

The authors have not responded to correspondence regarding this retraction.

## References

[1] Bi F, Chen C, Fu J, Yu L, Geng J (2021). Inhibiting proliferation and metastasis of osteosarcoma cells by downregulation of long non–coding RNA colon cancer–associated transcript 2 targeting microRNA–143 retraction in /10.3892/ol.2023.13665. Oncol Lett.

